# Application of high-throughput 5′P sequencing for the study of co-translational mRNA decay

**DOI:** 10.1016/j.xpro.2021.100447

**Published:** 2021-03-31

**Authors:** Yujie Zhang, Vicent Pelechano

**Affiliations:** 1 SciLifeLab, Department of Microbiology, Tumor and Cell Biology, Karolinska Institutet, Solna 171 65, Sweden

**Keywords:** Sequencing, RNAseq, Gene Expression

## Abstract

mRNA degradation is connected to the translation process up to the degree that 5′-3′ mRNA degradation follows the last translating ribosome. To study 5′-3′co-translational mRNA decay and the associated ribosome dynamics, here we present an improved high-throughput 5′P degradome RNA sequencing protocol (HT-5Pseq). We exemplify its application in *Saccharomyces cerevisiae*, but in principle, it could be applied to any other eukaryotic organism. HT-5Pseq is easy, scalable, and uses affordable duplex-specific nuclease-based rRNA depletion.

For complete details on the use and execution of this protocol, please refer to Zhang and Pelechano (2021).

## Highlights

• HT-5Pseq facilitates the investigation of 5′P mRNA degradome
• HT-5Pseq is an easy, scalable, and affordable protocol
• HT-5Pseq can be potentially applied in any eukaryotic organism

## Graphical abstract

**Figure:**
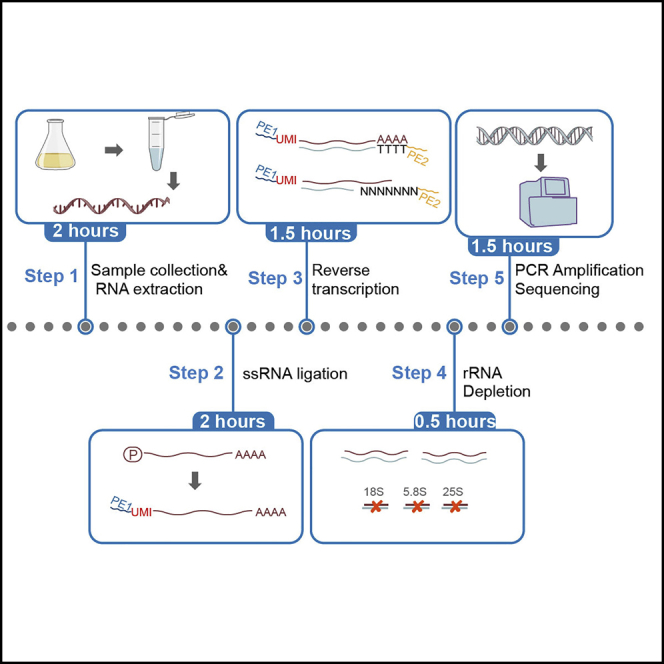

## Before you begin

### Overview of the protocol

**Figure 1:**
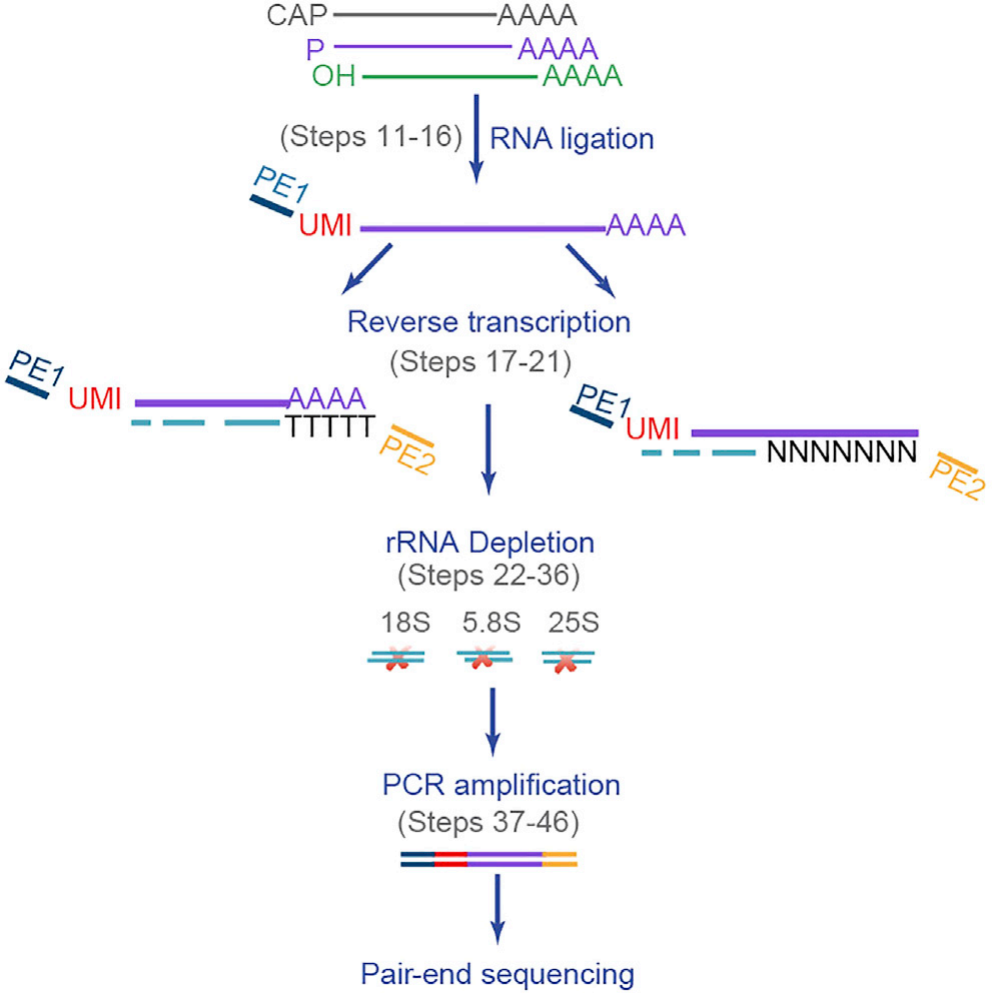
Detailed HT-5PSeq library workflow

HT-5Pseq captures in vivo 5′P mRNA degradation intermediates by ligating an RNA oligo to the exposed 5′P. After ligation, the RNA molecules are reverse-transcribed to cDNA using oligo-dT and random hexamer as primers. After cDNA library generation, cDNA generated from abundant rRNA molecules is removed using duplex-specific nuclease (DSN) and custom designed DNA probes. The remaining cDNA molecules are used as template for Illumina compatible sequencing library preparation (Figure 1).

### Prepare rRNA depletion probes mix

**Timing: 1 h**

1. Prepare the rRNA depletion probes mix:
  a. Mix equal volumes of the equimolar probes.
  b. Dilute probes mix from 200 μM to final 2 μM before use.

***Note:*** Here we use rRNA depletion probes (Table 1) designed for *S. cerevisiae*. However, it is possible to design rRNA depletion probes for the organism of interest.

**Table 1:** rRNA depletion oligos for *S. cerevisiae*

Oligo Name	Sequence 5′ to 3′
5.8S-1	TATTCCAGGGGGCATGCCTGTTTGAGCGTCATT
5S-1	ACCATACGCGAAACTCAGGTGCTGCAATCT
15S-1	GTAAAAAATTTATAAGAATATGATGTTGGTTCA
15S-2	TCAGATTAAGCGCTAAATAAGGACATGACACAT
18S-1	GTTT GACCTCAAATCAGGTAGGAGTACCCGCTGAACT
18S-2	AAACTTTCAACAACGGATCTCTTGGTTCTCG
18S-3	TATCTGGTTGATCCTGCCAGTAGTCATATGCTTGTC
18S-4	CAAAGATTAAGCCATGCATGTCTAAGTATAAGC
18S-5	CAGTGAAACTGCGAATGGCTCATTAAATCAG
18S-6	CATGGTATAACTGTGGTAATTCTAGAGCTAATACATGC
18S-7	ACCCTTTGGAAGAGATGTATTTATTAGAT
18S-8	TGATGATTCATAATAACTTTTCGAATCGCATGGC
18S-9	TGGCGATGGTTCATTCAAATTTCTGCCCTATCAACTT
18S-10	TCCTAATTCAGGGAGGTAGTGACAATAAATAACGA
18S-11	TACCTTAACGAGGAACAATTGGAGGGCAAGTC
18S-12	AGCAGCCGCGGTAATTCCAGCTCCAATAGC
18S-13	CGTGTACTGGATTTCCAACGGGGCCTTT
18S-14	TTGAAAAAATTAGAGTGTTCAAAGCAGGCGT
18S-15	TTGCTCGAATATATTAGCATGGAATAATAGAAT
18S-16	GTAATGATTAATAGGGACGGTCGGGGGCAT
18S-17	TGCCAAGGACGTTTTCATTAATCAAGAACGA
18S-18	ACTATGCCGACTAGGGATCGGGTGGTGTT
18S-19	CTCGGCACCTTACGAGAAATCAAAGTC
18S-20	AGGTCCAGACACAATAAGGATTGACAGATTGA
18S-21	AGTTGGTGGAGTGATTTGTCTGCTTAATTGC
18S-22	CGCTACACTGACGGAGCCAGCGAGTCTAACC
25S-1	GACCTCAAATCAGGTAGGAGTACCCGCTGAACT
25S-2	GGAAAAGAAACCAACCGGGATTGCCTTAG
25S-3	CTTGGAACAGGACGTCATAGAGGGTGAGAATC
25S-4	AGAGTCGAGTTGTTTGGGAATGCAGCTCTAAGT
25S-5	CAGTGATGGAAAGATGAAAAGAACTTTGAAAAG
25S-6	TCTAACGTCTATGCGAGTGTTTGGGTGTAAAAC
25S-7	CAAGAGGTGCACAATCGACCGATCCTGATGT
25S-8	ATCGAACCATCTAGTAGCTGGTTCCTGCCGAAGT
25S-9	ACGTAGAGTTAAGGTGCCGGAATACACGCTC
25S-10	ATATGGATTCTTCACGGTAACGTAACTGAATG
25S-12	TCCACAGGAAGGAATAGTTTTCATGCCAGGTCGTAC
25S-13	TCAAAGTGAAGAAATTCAACCAAGCGCGGGTAA
25S-14	GAGGGTGTAGAATAAGTGGGAGCTTCGGCGC
25S-15	ATAGGGAACGTGAGCTGGGTTTAGACCGTCG
25S-16	CTCTTCCTATCATACCGAAGCAGAATTCGG
25S-17	ACACAATATAGATGGATACGAATAAGGCGTC
25S-18	TACTGATGAATGTTACCGCAATAGTAATTG
25S-19	GAGGAACAGTTCATTCGGATAA
25S-20	AATCATTTGTATACGACTTAGATGTACAACGGG
21S-1	GTAAAAAGTAGAATAATAGATTTGAAATA
21S-2	TAGATTTAAAGAGATAATCATGGAGTATAA
21S-3	TATAAACTAATAAAGATCAGG
21S-4	CCGTAATGTAGACCGACTCAGGTATGTAAGTA

The mixed rRNA depletion oligos used in Step 27. The stock concentration is 200 μM. Working concentration is 2 μM.

## Key resources table

**Table:** 

REAGENT or RESOURCE	SOURCE	IDENTIFIER
**Chemicals, peptides, and recombinant proteins**		

Ethanol absolute ≥99.8%	VWR	20821.330
Glass beads, acid-washed	Sigma-Aldrich	G8772
Acid-Phenol:Chloroform, pH 4.5 (with IAA, 125:24:1)	Thermo Fisher Scientific	AM9722
dNTP set, 100 mM solution	Thermo Fisher Scientific	R0181
Phenol solution. Saturated with 0.1M citrate buffer, pH 4.3 ± 0.2	Sigma-Aldrich	P4682
Chloroform:isoamyl alcohol 24:1	Sigma-Aldrich	C0549
Sodium acetate buffer solution, pH 5.3	Sigma-Aldrich	S7899
Glycoblue coprecipitant (15 mg/mL)	Thermo Fisher Scientific	AM9515
Nuclease-free water, not DEPC treated	Thermo Fisher Scientific	AM9937
RiboLock RNase inhibitor 40 000U/mL	Thermo Fisher Scientific	EO0382
Turbo DNase kit	Thermo Fisher Scientific	AM1907
T4 RNA ligase 1	NEB	M0204L
SuperScript™ II Reverse Transcriptase	Thermo Fisher Scientific	18064071
Phusion®High-Fidelity PCR Master Mix	NEB	M0531S
AMPure XP	Beckman Coulter	A63881
RNAClean XP	Beckman Coulter	A63987
Duplex-specific nuclease	Evrogen	EA002

**Critical commercial assays**		

High Sensitivity DNA kit	Agilent	5067-4626
Qubit™ dsDNA HS Assay Kit	Thermo Fisher Scientific	Q32854
Qubit™ RNA HS assay kit	Thermo Fisher Scientific	Q32852

**Deposited data**		

The raw and processed sequencing data	This paper	GEO: GSE152375

**Experimental models: organisms/strains**		

*Saccharomyces cerevisiae* strains: BY4741:(MATa *his3Δ1 leu2Δ0 met15Δ0 ura3Δ0*)	NA	NA

**Oligonucleotides**		

See Tables 1 and 2	This paper	N/A

**Software and algorithms**		

*Fivepseq* package	GitHub	Nersisyan et al., 2020
bcl2fastq v2.20.0	Illumina	https://emea.support.illumina.com/sequencing/sequencing_software/bcl2fastq-conversion-software.html
Cutadapt	GitHub	https://github.com/marcelm/cutadapt/
UMI-tools	GitHub	Smith et al., 2017
STAR 2.7.0	GitHub	Dobin et al., 2013
DESeq2	Bioconductor	Love et al., 2014
Subread package	GitHub	Liao et al., 2014
IGV	https://igv.org	Thorvaldsdóttir et al., 2013
RStudio version 3.5.0	RStudio, Inc., Boston, MA	N/A

**Equipment**		

Refrigerated benchtop centrifuge	N/A	N/A
Thermo-block	N/A	N/A
Agilent Bioanalyzer 2100	Agilent	Bioanalyzer 2100
Chemical hood	N/A	N/A
Qubit Fluorometer	Invitrogen	QuBit 2.0
Magnet stand for PCR tubes	N/A	N/A
Vortex mixer	Scientific Industries	Vortex-Genie 2

## Materials and equipment

**Table:** LET lysis buffer

Reagent	Final Concentration
Tris, pH 8.0	25 mM
EDTA, pH 8.0	20 mM
LiCl	100 mM

Filter sterilize the LET lysis buffer. Use RNAse-free water. Possible for long term storage at 20°C–25°C.

## Step-by-step method details

### RNA extraction

**Timing: 2 h**

1. Obtain total RNA from sample of interest
  a. Harvest 1.5 mL cells from mid-log grown yeast (OD600 density ~ 0.6–0.8) using a standard bench microcentrifuge at 30 s, 8000 rpm at room temperature.
  b. Extract total RNA using phenol-chloroform extraction method.
    i. Set up the following tubes:
      Tube 1: 500 μL phenol:chloroform:IAA (125:24:1)
      Tube 2: 500 μL chloroform:IAA (24:1)
      Tube 3: 40 μL 3M NaOAc (made with nuclease-free H_2_O)
    ii. Add (approx. 200 μL) of glass beads and 150 μL LET in yeast cell pellet.
    iii. Add 150 μL phenol. Vortex at 20°C–25°C in vortex mixer for 2 min at top speed.
    iv. Add 250 μL nuclease-free H_2_O and 250 μL phenol:chloroform:IAA.
    v. Vortex in vortex mixer for additional 2 min followed by centrifugation at 4°C for 2 min at 14,000 × *g*.
    vi. Remove aqueous phase (approx. 450 μL) and add to Tube #1. Vortex 30 sec and spin 1 min at 14,000 × *g*.
    vii. Remove aqueous phase and add to Tube #2. Vortex 30 sec and spin 1 min at 14,000 rpm.
    viii. Remove aqueous phase (approx. 400 μL) and add to Tube #3 mix well. Add 1 mL 95% (vol/vol) ethanol, mix and place at −20°C/−80°C, 30 min.
    ix. Collect RNA by centrifugation at 4°C for 20 min at 14,000 × *g*.
    x. Wash pellet with 500 μL 70% (vol/vol) cold ethanol and re-centrifuged for 10 min.
    xi. Drain supernatant and air-dry pellet for ~ 3 min.
    xii. Resuspend pellet in 10 μL nuclease-free water.

**CRITICAL:** Phenol and Chloroform are acute toxic. Be careful when handling phenol and chloroform, always wear gloves, lab coat and perform manipulations following local safety regulations in fume hood.

**CRITICAL:** To avoid RNA degradation, perform RNA extraction with phenol:chloroform as fast as possible.

2. Check RNA quality by running a Bioanalyzer RNA gel or an agarose gel.

***Note:*** Any alternative approach producing high-quality RNA may be used instead.

### Removal of DNA from RNA samples

**Timing: 45 min**

Any contaminant DNA is removed from the sample.

3. Starting with 6 μg of total RNA, prepare the following mix and incubate the samples for 20 min at 37°C.

**Table:** 

Reagent	Final Concentration	Amount
TURBO DNAse buffer (10**×**)	1**×**	1 μL
TURBO DNAse (2 U/μL)	0.06 U/μL	0.3 μL
Ribolock (40 U/μL)	1.2 U/μL	0.3 μL
Sample RNA	0.7 μg/μL	8.4 μL
**Total**		**10 μL**

***Note:*** It is possible to lower starting material to 500 ng in total. However, low input material usually decreases library complexity and increase the PCR duplicates.

4. Add 2 μL of TURBO DNAse inactivation reagent and incubate 5 min at 20°C–25°C (tapping once in a while).
5. Centrifugate at 14,000 × *g* for 2 min at 20°C–25°C and transfer the supernatant to a clean precooled tube.

***Note:*** This pellet is normally quite loose, repeat the centrifugation if it is resuspended and avoid the carryover of any DNase inactivation reagent.

6. Ethanol precipitate the DNA-free RNA by adding 2.5 volumes (with respect to the sample volume) of 95% (vol/vol) ethanol, a 1/10 volume of 3 M sodium acetate, 1 μL of glycoblue. Mix sample by gently inverting and incubate it for minimum 30 min at −20°C/−80°C.

**Pause point:** The ethanol precipitation can be left 16–18 h at −20°C/−80°C.

7. Centrifugate at 14,000 × *g* for 30 min at 4°C to precipitate the RNA.
8. Wash the pellet with 500 μL of cold 70% (vol/vol).
9. Centrifugate 14,000 × *g* for 10 min at 4°C.
10. Remove the remaining ethanol, air-dry pellet for 3 min and resuspend it in 1.8 μL of RNAse-free water.

***Note:*** If the next step is the single-strand RNA ligation, RNA can be directly resuspended in ligation mix (step 11) and top up RNAse-free water to 10 μL .

**CRITICAL:** When handling with RNA samples, always keep them in RNase-free environment and place samples on ice.

### Single-strand RNA ligation

**Timing: 2 h**

5′ phosphate molecules are ligated with RNA oligos including unique molecular identifiers (UMI).

11. Prepare a 10 μL reaction mix with the components listed below:

**Table:** 

Reagent	Final Concentration	Amount
T4 RNA ligase buffer (10**×**)	1**×**	1 μL
rP5 _RND oligo (100 μM)	10 μM	1 μL
ATP (10 mM)	1 mM	1 μL
Ribolock (40 U/μL)	0.8 U/μL	0.2 μL
T4 RNA ligase 1 (10 U/μL)	1 U/μL	1 μL
PEG8000 (50%)	20%	4 μL
DNA-free RNA sample		1.8 μL
**Total**		**10 μL**

**CRITICAL:** Add PEG provided by T4 RNA ligase 1 in the end of the reaction mix, as it is sticky at high concentration.

12. Incubate sample at 25°C for 2 h.
13. Increase the sample volume with RNase-free water to 40 μL.
14. Purify the sample using 1.8**×** volumes of RNAClean XP beads, as described by the manufacturer’s instruction.
  a. Add 72 μL of RNAClean XP beads and mix sample by several times pipetting up and down.
  b. Incubate at 20°C–25°C for 5 mins until RNA bind to beads.
15. Place the PCR tubes at the magnets stand and wait till the solution is clear (~2 mins)
  a. Remove the supernatant.
  b. Wash beads twice with 200 μL of freshly made 70% (vol/vol) ethanol.
16. Remove the ethanol and let the beads slightly dry for 1 min.
  a. Elute samples in 12 μL RNase-free water.

***Note:*** avoid over drying the beads as that might result in sample loss.

### Reverse transcription

**Timing: 1.5 h**

cDNA library is transcribed by the defined ratio of oligo-dT and random hexamer.

17. Prepare a reaction mix as the components listed below:

**Table:** 

Reagent	Final Concentration	Amount
Total ligated RNA		10.6 μL
5PSeq RT oligo (20 μM)	1 μM	1 μL
5PSeq dT oligo (0.05 μM)	0.5 nM	0.2 μL
dNTPs (10 mM)	0.05 mM	0.1 μL
**Total**		**12.8 μL**

**CRITICAL:** This optimized ratio of oligo-dT and random hexamer increase coverage in the 3′ region of the gene. Altering the ratio of oligo-dT and random hexamer will lead to differences in the relative 5′/3′ coverage.

18. Denature the sample at 65°C for 5 min. Then place on ice directly.
19. To each tube, add 6.2 μL of mixture containing the following components:

**Table:** 

Reagent	Final Concentration	Amount
First-strand buffer (5**×**)	1**×**	4 μL
DTT (100 mM)	10 mM	2 μL
Ribolock (40 U/μL)	0.4 U/μL	0.2 μL
**Total**		**6.2 μL**

20. Add 1 μL of SuperScript II reverse transcriptase to each tube.
21. Incubate the sample at 25°C for 10 min, 42°C for 50 min and inactivate reaction at 70°C for 15 min.

***Note:*** Any commonly reverse transcriptase could be used instead of SuperScript II.

**Pause point:** Samples can be stored at −20°C.

### Remove template RNA

**Timing: 0.5 h**

Removing any excess of RNA, avoids that cDNA-RNA duplexes are degraded during duplex-specific nuclease (DSN) treatment.

22. Remove the template RNA by adding 100 mM NaOH (8 μL) to the sample (from step 21), and incubate at 65°C for 20 min.
23. Neutralized sample by adding 100 mM Tris-HCl, pH = 7.0 (8 μL).

***Note:*** It is also possible to remove excess RNA in cDNA-RNA duplex by using RNaseH.

24. Purify the sample using 1.8**×** volumes of Ampure XP beads, as described by the manufacturer’s instruction.
  a. Add 64.8 μL of Ampure XP beads and mix sample by several times pipetting up and down.
  b. Incubate at 20°C–25°C for 5 mins until samples bind to beads.
25. Place the PCR tubes at the magnets stand and wait till the solution is clear (~2 mins).
  a. Remove the supernatant.
  b. Wash beads twice with 200 μL of freshly made 70% (vol/vol) ethanol.
26. Remove the residual ethanol and allow the beads to slightly dry for 1 min. Elute in 8 μL of RNase-free water.

***Note:*** avoid over drying the beads as that might result in sample loss. If Ampure XP beads is not available, alternative beads such as MagSi magnetic beads (cat: MDKT00010075) can also be used.

### Duplex-specific nuclease (DSN) for rRNA depletion

**Timing: 0.5 h**

rRNA depletion is based on the designed DNA probes targeting ribosomal RNA (i.e., RDN18, RDN25, RDN5.8, RDN5).

27. Set up the following 16 μL reaction:

**Table:** 

Reagent	Final Concentration	Amount
Yeast rRNA depletion Probes (2 μM)	0.5 μM	4 μL
DSN master buffer (4**×**)	1**×**	4 μL
Sample from 26		8 μL
**Total**		**16 μL**

**CRITICAL:** The concentration of rRNA depletion probe mix is optimized for using 6 μg of total RNA as starting material. If the input RNA increases, the rRNA depletion probes should increase accordingly. The ratio between depletion probe mix and targeted molecules (cDNA) is around 2:1.

28. Denature sample for 2 min at 98°C using thermocycler.
29. Incubate the sample for 5 min at 68°C.
30. Add pre-warmed (2 min at 68°C) mix containing the following components:

**Table:** 

Reagent	Final Concentration	Amount
DSN enzyme (1 U/μL)	0.25 U/ μL	1 μL
DSN master buffer(4**×**)	1**×**	1 μL
H_2_O		2 μL
**Total**		**4μL**

**CRITICAL:** To avoid unspecific binding of probes to cDNAs during the treatment and thus unspecific degradation, it is critical to keep the reaction temperature at 68°C when mixing samples (from step 27 ) with DSN enzyme mix (from step 30). Pre-warm the reaction mix, using a separate PCR block and keep samples in the thermocycler when adding the pre-warmed mix.

31. Mix sample by pipetting several times and incubate for 20 min at 68°C in a thermocycler.
32. To inactivate DSN enzyme, add 20 μL of DSN stop solution (2**×**), mix contents and spin the tube briefly in a micro-centrifuge.
33. Incubate the sample for 10 min at 68°C temperature.
34. Purify the sample using 1.8**×** volumes of Ampure XP beads, as described by the manufacturer’s instruction.
  a. Add 72 μL of Ampure XP beads and mix sample by several times pipetting up and down.
  b. Incubate at 20°C–25°C for 5 mins until samples bind to beads.
35. Place the PCR tubes at the magnets stand and wait till the solution is clear (~2 min).
  a. Remove the supernatant.
  b. Wash beads twice with 200 μL of freshly made 70% (vol/vol) ethanol.
36. Remove the residual ethanol and allow the beads to slightly dry for 1 min. Elute in 9.6 μL of RNase-free water.

**Note:** avoid over drying the beads as that might result in sample loss.

### Library PCR amplification

**Timing: 1.5 h**

PCR is used to generate an Illumina compatible sequencing library

37. To amplify the library by PCR, prepare the following mix:

**Table:** 

Reagent	Final Concentration	Amount
Phusion high-fidelity PCR master-mix (2**×**)	1**×**	10 μL
Illumina compatible PE1.0 (10 μM)	0.1 μM	0.2 μL
Illumina compatible PE2_MPX (10 μM)	0.1 μM	0.2 μL
Sample (from step 36)		9.6 μL
**Total**		**20 μL**

**CRITICAL:** To increase the sequencing complexity per sample and enable multiplexing, we use different oligos for each sample (PE2_MPX, Table 2). This also applies to dual index barcoding with PE1 compatible multiplexing oligos. To better multiplex samples and avoid base calling issues afterwards, please use different barcodes for sequencing base composition balance. For more detailed information related to base composition balance, please check on Illumina website.

***Note:*** Any good quality used thermostable DNA polymerase used for sequencing library preparation could be used instead.

38. Run the following PCR program:

**Table:** 

PCR Cycling Conditions			
Steps	Temperature	Time	Cycles
Initial Denaturation	98°C	30 sec	1
Denaturation	98°C	20 sec	14–18 cycles
Annealing	65°C	30 sec	
Extension	72°C	30 sec	
Final extension	72°C	7 min	1
Hold	4°C

39. Bring the volume to 100 μL with nuclease-free water.
40. Add 0.7**×** Ampure XP beads to the sample to bind the long DNA molecules. For 100 μL of sample, add 70 μL of beads and mix by pipetting. Incubate at 20°C–25°C for 5 mins.
41. Place the PCR tube on the magnets stand and wait till the solution is clear. Transfer the supernatant to a new tube.

**CRITICAL:** At this moment, the molecules of interest are in the supernatant, not on the beads.

42. Add with 0.2**×** Vol. (respect to the original sample volume) of Ampure XP beads (20 μL respect to 100 μL) to the recovered supernatant from step 41. Mix the sample by pipetting up and down and incubate at 20°C–25°C for 5 mins.
43. Place the PCR tubes at the magnets stand and wait till the solution is clear (~2 mins).
  a. Remove the supernatant
  b. Wash beads twice with 200 μL of freshly made 70% (vol/vol) ethanol.
44. Remove the residual ethanol and allow the beads to slightly dry for 1 min. Elute in 10 μL in water or EB buffer (10 mM Tris-HCl, pH 8.0).
45. Measure the final library concentration by Qubit using the dsDNA HS assay kit and check the size distribution by Bioanalyzer.
46. Sequence the libraries using pair-end 75 cycles Illumina NextSeq 500.

***Note:*** For pair-end 75 cycles sequencing in this case, using 60 bp for read1 and 15 bp for read2. Read2 will identify the molecule primed by either oligo-dT or random hexamer. In general, we recommend at least 6 million raw reads per yeast sample. Any alternative Illumina platform could be used instead of a NextSeq 500. Read sequencing length can be altered depending on the complexity of the genome of interest and the ability to uniquely map reads to the genome.

**Table2:** Oligonucleotides used for HT-5Pseq library preparation

Primer name	Purpose	Sequence (5′-3′)
RNA_rP5_RND	To add UMI and common sequence to the RNAs (step 11)	rCrUrUrUrCrCrCrUrArCrArCrGrArCrGrCrUrCrUrUrCrCrGrArUrCrU rNrNrNrNrNrNrNrN
5Pseq-dT	To do reverse transcription by using oligo-dT and add common sequence to the libraries (step 17)	GTGACTGGAGTTCAGACGTGTGCTC TTCCGATCT TTTTTTTTTT
5Pseq-RT	To do reverse transcription by using random hexamer and add common sequence to the libraries (step 17)	GTGACTGGAGTTCAGACGTGTGCTC TTCCGATCT NNNNNN
Illumina compatible PE1.0	To generate library by PCR (step 37)	ATGATACGGCGACCACCGAGATCTACACTCTTTCCCTACACG ACGCTCTTCCGATC∗T
PE2_MPX_01	To add multiplex barcode by PCR (step 37)	CAAGCAGAAGACGGCATACGAGAT**CGTGAT**GTGACTGGAGT TCAGACGTGTGCTCTTCCGATC∗T
PE2_MPX_02	To add multiplex barcode by PCR (step 37)	CAAGCAGAAGACGGCATACGAGAT**ACATCG**GTGACTGGAGT TCAGACGTGTGCTCTTCCGATC∗T
PE2_MPX_03	To add multiplex barcode by PCR (step 37)	CAAGCAGAAGACGGCATACGAGAT**GCCTAA**GTGACTGGAGT TCAGACGTGTGCTCTTCCGATC∗T
PE2_MPX_04	To add multiplex barcode by PCR (step 37)	CAAGCAGAAGACGGCATACGAGAT**TGGTCA**GTGACTGGAGT TCAGACGTGTGCTCTTCCGATC∗T
PE2_MPX_05	To add multiplex barcode by PCR (step 37)	CAAGCAGAAGACGGCATACGAGAT**CACTGT**GTGACTGGAGT TCAGACGTGTGCTCTTCCGATC∗T
PE2_MPX_06	To add multiplex barcode by PCR (step 37)	CAAGCAGAAGACGGCATACGAGAT**ATTGGC**GTGACTGGAGT TCAGACGTGTGCTCTTCCGATC∗T
PE2_MPX_07	To add multiplex barcode by PCR (step 37)	CAAGCAGAAGACGGCATACGAGAT**GATCTG**GTGACTGGAGT TCAGACGTGTGCTCTTCCGATC∗T
PE2_MPX_08	To add multiplex barcode by PCR (step 37)	CAAGCAGAAGACGGCATACGAGAT**TCAAGT**GTGACTGGAGT TCAGACGTGTGCTCTTCCGATC∗T
PE2_MPX_09	To add multiplex barcode by PCR (step 37)	CAAGCAGAAGACGGCATACGAGAT**CTGATC**GTGACTGGAGT TCAGACGTGTGCTCTTCCGATC∗T
PE2_MPX_10	To add multiplex barcode by PCR (step 37)	CAAGCAGAAGACGGCATACGAGAT**AAGCTA**GTGACTGGAGT TCAGACGTGTGCTCTTCCGATC∗T
PE2_MPX_11	To add multiplex barcode by PCR (step 37)	CAAGCAGAAGACGGCATACGAGAT**GTAGCC**GTGACTGGAGT TCAGACGTGTGCTCTTCCGATC∗T
PE2_MPX_12	To add multiplex barcode by PCR (step 37)	CAAGCAGAAGACGGCATACGAGAT**TACAAG**GTGACTGGAGT TCAGACGTGTGCTCTTCCGATC∗T
PE2_MPX_13	To add multiplex barcode by PCR (step 37)	CAAGCAGAAGACGGCATACGAGAT**TTGACT**GTGACTGGAGT TCAGACGTGTGCTCTTCCGATC∗T
PE2_MPX_14	To add multiplex barcode by PCR (step 37)	CAAGCAGAAGACGGCATACGAGAT**GGAACT**GTGACTGGAGT TCAGACGTGTGCTCTTCCGATC∗T
PE2_MPX_15	To add multiplex barcode by PCR (step 37)	CAAGCAGAAGACGGCATACGAGAT**TGACAT**GTGACTGGAGT TCAGACGTGTGCTCTTCCGATC∗T
PE2_MPX_16	To add multiplex barcode by PCR (step 37)	CAAGCAGAAGACGGCATACGAGAT**GGACGG**GTGACTGGAGT TCAGACGTGTGCTCTTCCGATC∗T
PE2_MPX_17	To add multiplex barcode by PCR (step 37)	CAAGCAGAAGACGGCATACGAGAT**CTCTAC**GTGACTGGAGT TCAGACGTGTGCTCTTCCGATC∗T
PE2_MPX_18	To add multiplex barcode by PCR (step 37)	CAAGCAGAAGACGGCATACGAGAT**GCGGAC**GTGACTGGAGT TCAGACGTGTGCTCTTCCGATC∗T
PE2_MPX_19	To add multiplex barcode by PCR (step 37)	CAAGCAGAAGACGGCATACGAGAT**TTTCAC**GTGACTGGAGT TCAGACGTGTGCTCTTCCGATC∗T
PE2_MPX_20	To add multiplex barcode by PCR (step 37)	CAAGCAGAAGACGGCATACGAGAT**GGCCAC**GTGACTGGAGT TCAGACGTGTGCTCTTCCGATC∗T
PE2_MPX_21	To add multiplex barcode by PCR (step 37)	CAAGCAGAAGACGGCATACGAGAT**CGAAAC**GTGACTGGAGT TCAGACGTGTGCTCTTCCGATC∗T
PE2_MPX_22	To add multiplex barcode by PCR (step 37)	CAAGCAGAAGACGGCATACGAGAT**CGTACG**GTGACTGGAGT TCAGACGTGTGCTCTTCCGATC∗T
PE2_MPX_23	To add multiplex barcode by PCR (step 37)	CAAGCAGAAGACGGCATACGAGAT**CCACTC**GTGACTGGAGT TCAGACGTGTGCTCTTCCGATC∗T
PE2_MPX_24	To add multiplex barcode by PCR (step 37)	CAAGCAGAAGACGGCATACGAGAT**GCTACC**GTGACTGGAGT TCAGACGTGTGCTCTTCCGATC∗T
PE2_MPX_25	To add multiplex barcode by PCR (step 37)	CAAGCAGAAGACGGCATACGAGAT**ATCAGT**GTGACTGGAGT TCAGACGTGTGCTCTTCCGATC∗T
PE2_MPX_26	To add multiplex barcode by PCR (step 37)	CAAGCAGAAGACGGCATACGAGAT**GCTCAT**GTGACTGGAGT TCAGACGTGTGCTCTTCCGATC∗T
PE2_MPX_27	To add multiplex barcode by PCR (step 37)	CAAGCAGAAGACGGCATACGAGAT**AGGAAT**GTGACTGGAGT TCAGACGTGTGCTCTTCCGATC∗T
PE2_MPX_28	To add multiplex barcode by PCR (step 37)	CAAGCAGAAGACGGCATACGAGAT**CTTTTG**GTGACTGGAGT TCAGACGTGTGCTCTTCCGATC∗T
PE2_MPX_29	To add multiplex barcode by PCR (step 37)	CAAGCAGAAGACGGCATACGAGAT**TAGTTG**GTGACTGGAGT TCAGACGTGTGCTCTTCCGATC∗T
PE2_MPX_30	To add multiplex barcode by PCR (step 37)	CAAGCAGAAGACGGCATACGAGAT**CCGGTG**GTGACTGGAG TTCAGACGTGTGCTCTTCCGATC∗T

∗ refers to S-linkage between the two bases r refers to a RNA base. Barcodes are identified in bold black. Sequences are represented in 5′ to 3′orientation. # Oligonucleotide sequences © 2006–2021 Illumina, Inc. All rights reserved.

## Expected outcomes

**Figure 2:**
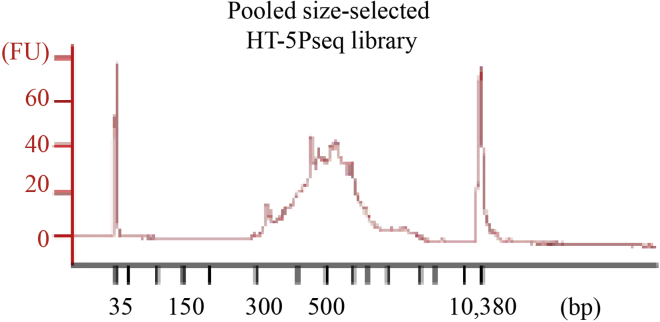
Example of a size-selected HT-5PSeq pool libraries (step 45)

**Figure 3:**
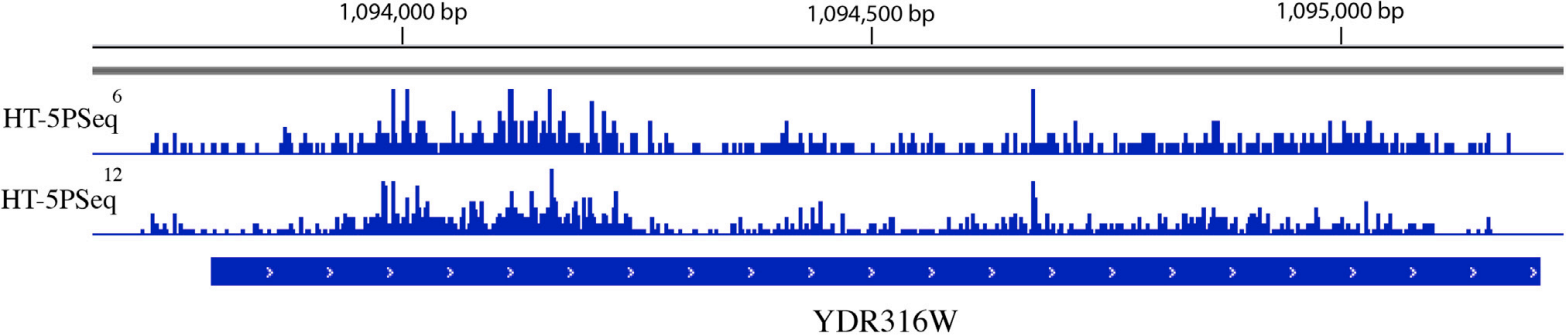
Example of HT-5PSeq reads visualized by IGV (Thorvaldsdóttir et al., 2013) for *S. cerevisiae* Two Biological replicates are shown.

**Figure 4:**
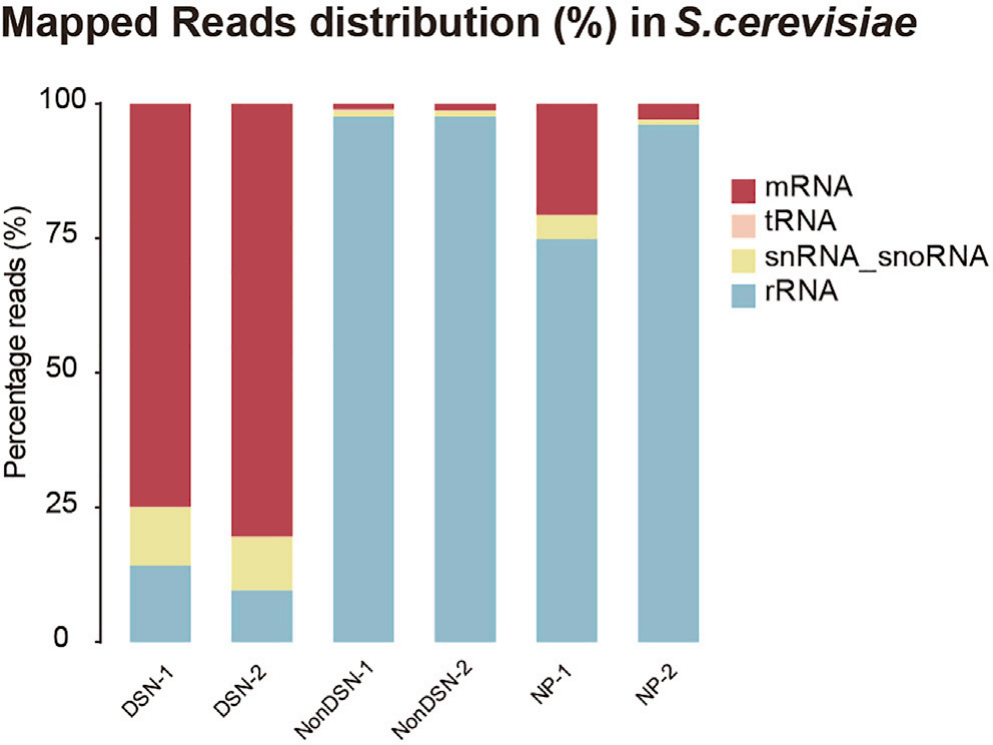
Distribution of mRNA, rRNA reads in *S. cerevisiae* HT-5Pseq after rRNA depletion NonDSN refers to control libraries omitting DSN rRNA depletion. NonProbe refers to libraries treated with DSN but omitting the depletion oligos (Data from (Zhang and Pelechano, 2021)). Two biological replicates are shown.

**Figure 5:**
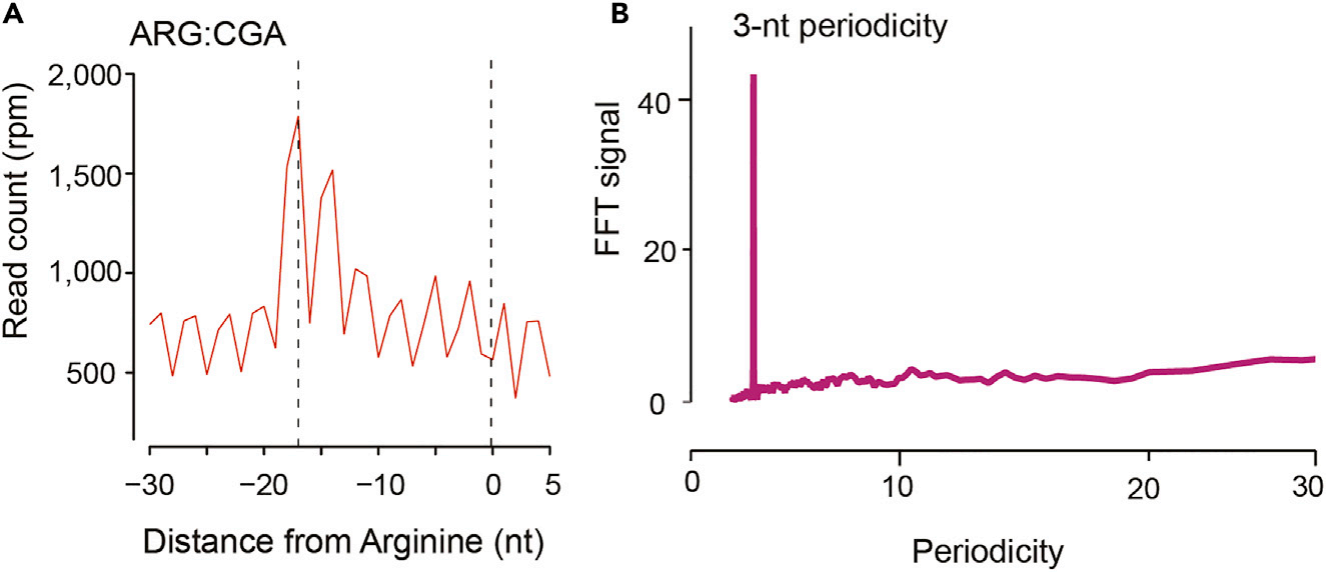
HT-5Pseq reveals ribosome dynamics at codon resolution (Data from (Zhang and Pelechano, 2021)) (A) Metagene analysis for 5 ´P read coverage relative to arginine (CGA). Dotted lines at -17 corresponding to the expected 5′ end of protected ribosome located at A site. (B) 3-nt periodicity shown in 5′P read by Fourier transform calculation.

This protocol will generate sequencing libraries of 5′P mRNA degradation intermediates, detailed workflow is shown in Figure 1. In the final HT-5Pseq library, the average size is expected to be around 450 bp, including 150 bp Illumina adapter sequences (Figure 2). The expected concentration of library can be 0.5–2 ng/μL. The sequencing depth required will depend on the library complexity and analysis requirement. By mapping HT-5Pseq reads to the reference genome, the expected results are as following: 1) reads coverage is distributed along the whole mRNA regions (Figure 3); 2) rRNA contamination of HT-5Pseq library is less than 12%–20% (Figure 4); 3) A clear 3-nt pattern can be observed with respect to specific codons, including start and stop codon at metagene level (Figure 5); 4) Codon-specific/amino acid specific pausing can be extracted with respect to specific codons.

## Quantification and statistical analysis

Here we provide a potential bioinformatic pipeline for 5′Pseq data.

• De-multiplex raw data using the indexing information: Using bcl2fastq (v2.20.0) for base-calling. We recommend allowing 1 mismatch in index 1 and 1 mismatch in index2.
• Trim sequencing adaptor: Use cutadapt V1.16 to trim sequencing adapter (-a AGATCGGAAGAGCACACGTCTGAACTCCAGTC).
• Extract UMI: Use UMI-tools (v0.5.4) to extract 8-nt random barcodes on the 5′ ends of reads. These UMI information will be used to remove PCR duplicates.
• Align sequencing reads: Use star/2.7.0 (Dobin et al., 2013) to align 5′-end reads to reference genome (SGD R64-1-1 for *S. cerevisiae* genome). For mapping the 5′-ends reads to the genome, we recommend using the parameter --alignEndsType Extend5pOfRead1 to exclude soft-clipped bases on the 5′end.
• Remove PCR duplicates: Use UMI-tools (v0.5.4) to remove duplicated 5′ ends of read introduced by PCR during library preparation.
• Quantify transcripts: Use Subread package (featureCounts) (Liao et al., 2014) to count mRNA, tRNA, rRNA and snRNA and snoRNA transcripts. Use DESeq2 packages from R and Bioconductor (Love et al., 2014) to perform differential gene expression analysis.
• Analysis 5′ ends positions: Use *Fivepseq* package to map 5′ ends with respect to start, stop codon and codons at metagene level (Nersisyan et al., 2020).

## Limitations

Although HT-5PSeq offers high quality degradome information at a fraction of the costs and with significantly decreased hands-on time in comparison with standard 5PSeq (Pelechano et al., 2015), this approach has several limitations that need to be accounted for.

Firstly, the main limitation is that HT-5Pseq approach focus on the subpopulation of 5′ end of mRNA undergoing decay. Therefore, any exposed 5′P end of molecule can be captured independent of their relationship with co-translation degradation process.

Next, HT-5Pseq measures the kinetics competition of 5′-3′ degradation machinery and ribosome, therefore we do not recommend this approach to directly measure absolute translation rates. However, we have shown that the last translating ribosome during co-translational decay can infer the general ribosome dynamics (Pelechano et al., 2015).

Thirdly, as the abundance of 5′P end molecule depends on both translation and mRNA stability, any factor involved in those process can affect the observed 5′P seq profile. For example, when investigating mRNA degradation profiles in *xrn1Δ* the ribosome associated 3-nt pattern is greatly decreased (Pelechano et al., 2015). In *xrn1Δ* the observed 5′P profiles reflects a combination of transcription start site mapping (as expected from the exposed 5′P after decapping) complemented by other endonucleolitica cleavages events. In addition, HT-5Pseq libraries may vary in library complexity as a result of the variation of fractions on mRNA degradation intermediates present in a sample in respect to the total RNA. For example, HT-5PSeq libraries from *xrn1Δ* cells are in general more complex, as 5′P mRNA degradation intermediates are not efficiently removed and thus represent a higher proportion of the total RNA population. To control for this, we add UMI during the RNA ligation step. If a lower fraction of mRNA degradation intermediates is expected, we recommend increasing the amount of total RNA starting material.

## Troubleshooting

### Problem 1

RNA degradation

### Potential solution

1) When handling with RNA samples, always keep RNA on the ice.
2) Check the RNA integrity of RNA extraction.
3) Use RNase inhibitor during the protocol (steps 11 and 19) and aliquot RNase-free reagents.
4) Perform RNA extraction with phenol-chloroform (step 1) as fast as possible.

### Problem 2

Low yield DNA library (step 45)

### Potential solution

1) If starting RNA is less, increase the starting RNA amount.
2) Inefficient removal of RNA template (step 22), this will loss the cDNA library after DSN treatment. Optimize RNA removal steps (step 22).
3) Increase few PCR cycles in PCR amplification steps (step 38). Final PCR cycles should be less than 20 cycles.
4) For bead cleanup, do not over dry the beads. That might lead to sample loss.

### Problem 3

Large number of rRNA reads

### Potential solution

1) DNA leftover in RNA sample can potential saturate DSN enzymatic activity (step 27). This may decrease the rRNA depletion efficiency. Perform DNase treatment to RNA samples (step 3).
2) Optimize DSN for rRNA depletion if using custom depletion probes (step 27–36). Optimize mix annealing temperature (steps 29–30) based on the melting temperature of newly designed probes. Mix samples with probes by pipetting and keep them on the thermocycle at the selected temperature.

### Problem 4

Large number of PCR duplicates

### Potential solution

3) Increase the input RNA material to increase the complexity.
4) Decrease the final PCR cycles (from step 38) that will increase the useful reads.

### Problem 5

Biased 5′P reads to 3′ end

### Potential solution

5) If the 5′P reads biased towards to 3′, decrease the usage of oligo-dT in reverse-transcription (step 17) to get more homogenous distribution profile.

## Resource availability

### Lead contact

Further information and requests for resources and reagents should be directed to and will be fulfilled by the lead contact, Vicent Pelechano (vicente.pelechano.garcia@ki.se) .

### Materials availability

This study did not generate new unique materials nor reagents.

### Data and code availability

The raw and processed sequencing data are deposited at GEO with accession number GSE152375.
